# New-Onset Polymyalgia Rheumatica Complicated by Giant Cell Arteritis Following COVID-19 Infection

**DOI:** 10.7759/cureus.41951

**Published:** 2023-07-16

**Authors:** Divya Mamootil

**Affiliations:** 1 Internal Medicine, Ascension Saint Agnes Hospital, Baltimore, USA

**Keywords:** polymyalgia rheumatica, chronic steroid use, tocilizumab, prednisone, giant cell arteritis, pmr, gca, giant cell arteritis with polymyalgia rheumatica, covid-19, sars-cov-2

## Abstract

A 68-year-old female with a past medical history significant for tophaceous gout presented with pain and stiffness in her bilateral shoulders and hip joints for about two weeks after testing positive for COVID-19. Her laboratory results showed an elevated erythrocyte sedimentation rate (ESR) of 74 mm/h and C-reactive protein (CRP) of 25 mg/L. She showed clinical improvement in her symptoms after steroid therapy and was diagnosed with polymyalgia rheumatica (PMR). Despite prompt treatment with steroids, she continued to have persistent joint pain. Also, she developed new bilateral temporal artery tenderness, headaches, blurry vision, and jaw claudication concerning giant cell arteritis (GCA). She was admitted to the hospital for high-dose pulsed IV methylprednisolone and discharged with a steroid taper along with tocilizumab injections. Her symptoms improved rapidly, and she continued to follow up with rheumatology while continuing low-maintenance doses of prednisone. Although the association between PMR and GCA is well-known, the time it takes to reach disease remission, the rate of relapse, and the length of steroid treatment are variable. There are a few COVID-19-associated cases of PMR and GCA; however, the timeline and pathophysiology of this association remain an area for further investigation.

## Introduction

Polymyalgia rheumatica (PMR) is an inflammatory rheumatic disease causing bilateral pain and stiffness in the shoulder and hip joints. Giant cell arteritis (GCA), on the other hand, is a similar inflammatory disease affecting the vasculature. It presents with symptoms such as headaches, scalp tenderness, jaw pain, vision loss, and may lead to long-term complications like aortic aneurysms. Both of these diseases commonly present in individuals over the age of 50 [1]. Around 40-60% of GCA patients exhibit PMR symptoms, while approximately 20% of PMR patients display GCA symptoms [2]. There are no specific laboratory tests for the diagnosis of PMR and GCA; however, both conditions initially show increased inflammatory markers such as erythrocyte sedimentation rate (ESR) and C-reactive protein (CRP). The etiology of these diseases is not well-known; however, it is thought that an immune-mediated pathway might trigger an auto-inflammatory response, independent of other autoimmune risk factors [3].

## Case presentation

A 68-year-old African American female with past medical history of hypertension, chronic obstructive pulmonary disease (COPD), thyroid nodules and tophaceous gout (taking allopurinol and colchicine), presented to the outpatient Rheumatology clinic with new onset pain and stiffness in the bilateral shoulders and hips for 1 month. She had difficulty getting up from a chair and over-the-head movements. She denied any muscle weakness, or numbness/tingling sensations. She denied scalp tenderness, jaw cramping, or vision loss at the time. She was given a Medrol (methylprednisolone) dose pack with resolution of her symptoms. Of note, she was recently diagnosed with COVID -19 for the first time about two weeks prior to her symptom onset, and had received Paxlovid (Nirmatrelvir/ritonavir) at that time. She had only received two SARS-COV-2 vaccines about two years prior. 

On the physical exam, vital signs showed blood pressure of 146/82 mmhg, heart rate 56 beats per minute, temperature 98.4 degrees Fahrenheit, and pulse oximetry 100% on room air. Pupils were equal and reactive to light with intact extraocular movements. She had no tenderness to palpation on the scalp, temporal arteries, or jaw. Her temporal arteries were +2 bilaterally, without carotid or subclavicular bruits. There was no synovitis on the exam. She had normal muscle strength 5/5 in the shoulder and hips, however her range of motion was limited in both. There was tophaceous gout in the bilateral 1st metatarsophalangeal (MTP) joints.

Laboratory results revealed white blood cell (WBC) of 9.8 K/uL (reference 4-11 K/uL), hemoglobin of 13.1 g/dL (reference 12-15 g/dL), and creatinine of 1.1 mg/dL (reference 0.5-1 mg/dL). Erythrocyte sedimentation rate (ESR) was 74 mm/h (reference 0-30 mm/h) and C-reactive protein (CRP) was 25 mg/L (reference <1 mg/L) (Table 1). Rheumatoid factor (RF) was <10 IU/mL (reference <14 IU/mL), Cyclic citrulline peptide antibody (CCP) was 2 (reference 0-19 units negative), and Nuclear antibody (ANA) screen was <1:80 (reference <1:80 negative) (Table 2).

**Table 1:** Inflammatory markers (blood).

Inflammatory markers	Value (reference range)
Erythrocyte sedimentation rate (ESR)	74 mm/h (0-30 mm/h)
C-reactive protein (CRP)	25 mg/L (<1 mg/L)

**Table 2:** Serum immunology panel.

Serum Immunology Panel	Value (reference range)
Nuclear antibody (ANA) screen	<1:80 (<1:80 negative)
Rheumatoid factor (RF)	<10 IU/mL (<14 IU/mL negative)
Cyclic citrulline peptide antibody (CCP)	2 (0-19 units negative)

**Figure 1:**
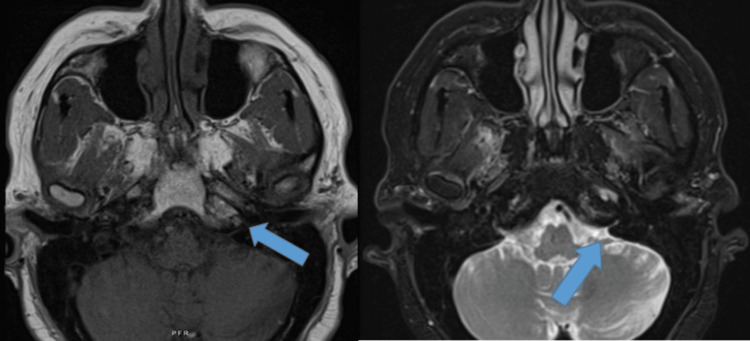
Coronal T1 and T2-weighted brain MRI showing a small focus of extra-axial restricted diffusion in the left cerebellopontine angle cistern with associated enhancement and increased enhancement along the adjacent tentorium.

**Figure 2:**
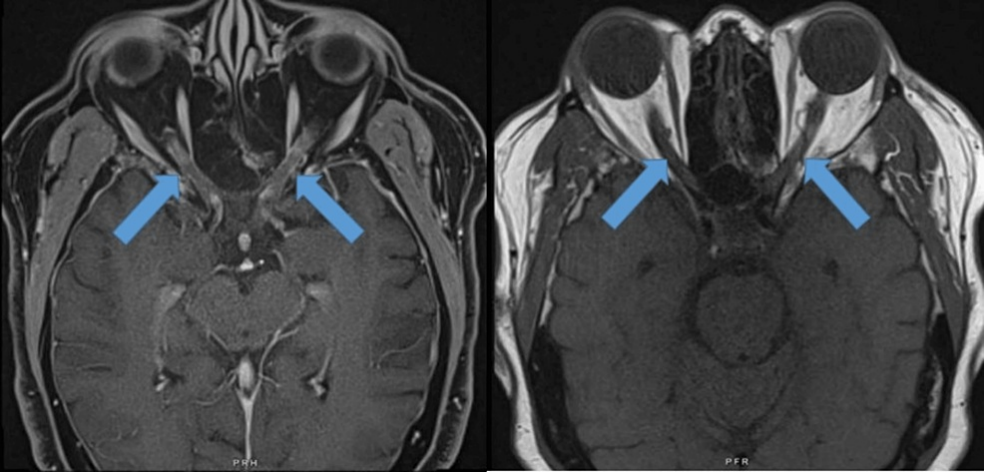
Axial T1 and T2-weighted orbital MRI with fat suppression showing the mildly increased signal intensity of bilateral optic nerves and subtle optic nerve enhancement.

Ultrasound examination of the bilateral temporal arteries was negative for a halo sign. Similarly, an ultrasound examination of the left shoulder revealed fluid in the subacromial bursa and Doppler activity around the biceps tendon, consistent with inflammation. She was diagnosed with PMR and prescribed a prolonged steroid taper starting at 20 mg daily for two weeks, with the goal to decrease the dose by 2.5 mg per week until 10 mg daily. On her six-week follow-up visit with Rheumatology, she complained about occipital headaches, as well as new jaw locking and tenderness while chewing food. She denied any vision loss, pain, or diplopia at the time. Since she was already taking prednisone, it was decided that obtaining a temporal artery biopsy or using ultrasound would be of low diagnostic yield. Hence, she was started on a high-dose regimen of prednisone, 60 mg daily (1 mg/kg daily), with a plan to taper it down to 20 mg daily over a six-week period following the Giant-Cell Arteritis Actemra (GiACTA) trial protocol. Additionally, Omeprazole 20 mg daily was added for stress ulcer prophylaxis. One week following her office visit, she presented to the ER with complaints of persistent headache and jaw pain, as well as new right eyelid swelling and bilateral blurry vision that was worse in the right eye. Her jaw pain was worse with chewing food, preventing her from eating. Her headaches had become tension-like, with bilateral temporal artery swelling and tenderness. On the physical exam, she had mild swelling and tenderness to palpation over the right zygomatic arch. Her visual acuity was 20/20 in the left eye and 20/40 in the right eye, with intact extraocular muscles. A repeat ESR test showed 14 mm/h (within the reference range of 0-30 mm/h), and her CRP level was 0.5 (within the reference range of <1 mg/L). A CT scan of the head showed intracranial atherosclerosis with no acute infarct or hemorrhage present. She was diagnosed with Giant Cell Arteritis (GCA) and admitted to the inpatient service with a plan to continue a daily dosage of prednisone at 60 mg. Vascular surgery was consulted regarding a potential temporal artery biopsy; however, it was decided not to pursue this avenue. The patient had already been on steroids, which could affect the histopathology, and she declined further action. An MRI of the brain, face, and orbit revealed a small focus of extra-axial restricted diffusion in the left cerebellopontine angle cistern, expectedly at the location of the 5th cranial nerve. There was associated enhancement, increased enhancement along the adjacent tentorium, and mild enhancement of the bilateral optic nerves, which could be related to inflammation (Figures 1-2).

Upon Ophthalmology examination, a cotton wool spot was observed in her right eye, and she was diagnosed with optic neuritis in the setting of GCA. She was treated with IV methylprednisolone 250 mg every six hours for 12 doses, followed by a prednisone taper starting with 80 mg daily upon discharge for two weeks. During her outpatient follow-up with Rheumatology, she was started on tocilizumab (Actemra) 162 mg subcutaneous injections weekly, along with prednisone 60 mg daily for two weeks with the plan to taper over a few months. The patient responded well to this treatment regimen, and over the next six months, she was able to taper down to 2.5 mg of prednisone daily.

## Discussion

PMR

Diagnosis of PMR is mainly based on clinical presentation; however, it is always helpful to check inflammatory markers such as ESR and CRP, which are often, but not always, elevated in these patients. Imaging such as ultrasound or X-ray of the joints can be valuable in diagnosis, as they may show fluid collections or Doppler activity, which can be signs of inflammation. The American College of Rheumatology (ACR) classification of PMR is applied to patients over the age of 50 with new onset of symptoms (<12 weeks) and the following criteria: morning stiffness >45 minutes (+2), hip pain or limited range of motion (+1), absence of RF or anti-CCP antibodies (+2), and absence of other joint involvement (+1) [4]. Without ultrasound, a total score of 0-6 is given, with a score of 4 and above confirming the diagnosis of PMR. With the use of ultrasound, two additional criteria are added: at least one shoulder with subdeltoid bursitis, biceps tenosynovitis and/or glenohumeral synovitis, and at least one hip with synovitis and/or trochanteric bursitis (+1), both shoulders with subdeltoid bursitis, biceps tenosynovitis and/or glenohumeral synovitis (+1) [4]. This would give a total score of 0-8, with a score of 5 and above confirming the diagnosis. A prompt response to low-dose steroid therapy of less than 20 mg per day is also a factor used in diagnosing PMR [3]. The Polymyalgia Rheumatica Activity Score (PMR-AS) can be used to monitor disease activity. This score measures the duration of morning stiffness in minutes, the ability to elevate the upper limbs (with 3 signifying no ability, 2 below the shoulder girdle, 1 up to the shoulder girdle, and 0 above the shoulder girdle), a physician's global assessment (with a score from 0 to 10), the patient's subjective pain level (also scored from 0 to 10), and the CRP level in mg/dL [2]. A PMR-AS score higher than 17 represents high disease activity, a score less than 7 represents low disease activity, and a score less than 1.5 signifies remission [2].

GCA

GCA is a type of large vessel vasculitis affecting both cranial and extracranial arteries [2]. According to the 2022 ACR recommendations, diagnosis of GCA requires an age of 50 or above at disease onset in all patients, along with a total score of 6 or more points from the following criteria: elevated ESR equal to or greater than 50 mm/h or C-reactive protein equal to or greater than 10 mg/dL (+3), positive temporal artery biopsy or temporal artery halo sign on ultrasound (+5), sudden vision loss (+3), and morning stiffness in the shoulders/neck, jaw or tongue claudication, scalp tenderness, temporal artery abnormality on the vascular exam, bilateral axillary involvement on image, or fluorodeoxyglucose (FDG) PET-CT activity in the aorta (+2 each) [5]. Various imaging findings can be used to diagnose GCA, including ultrasound, PET-CT, MRA, or CTA. An important caveat to imaging is that the diagnostic yield of imaging will decrease with steroid therapy; however, treatment should not be delayed to obtain imaging [6]. GCA can progress to vision loss in about 15-35% of patients, ranging from symptoms of diplopia and amaurosis fugax to blindness [2]. Since the risk of permanent vision loss is high, it is imperative to start high-dose steroid treatment as soon as possible. 

Treatment options

Glucocorticoids are the first-line therapy for both PMR and GCA. PMR is usually very responsive to low doses of prednisone, around 10-20 mg daily [1]. In GCA, patients are usually treated with IV methylprednisolone 250-1000 mg daily for three days, followed by a steroid taper with an initial dose of 40-60 mg daily and a goal of reaching <5 mg daily in a year [2]. Treatment may take months to years and will vary from patient to patient. Unfortunately, most of these patients require several months of therapy. They are at high risk of steroid-related adverse effects, including osteoporosis, weight gain, hyperglycemia, hypertension, dyslipidemia, and increased susceptibility to infections [2]. For steroid-sparing treatment options, methotrexate has been used in some patients, although studies have been limited [6]. One of the more novel therapies that have been used with success is tocilizumab, an interleukin 6 (IL-6) inhibitor, as demonstrated in the GiACTA trial. This trial gave tocilizumab injections weekly or every other week to GCA patients with a concurrent 26-week or 52-week prednisone taper [7]. This trial showed that over 50% of patients in the tocilizumab groups had sustained remission at 52 weeks compared to less than 20% of patients in the placebo group at either 26 weeks or 52 weeks [7]. The trial also revealed significantly fewer adverse effects in the tocilizumab group than in the placebo group, regardless of the duration of steroid treatment [7]. Another multi-center trial in France investigated the use of tocilizumab and prednisone taper for PMR patients with chronic daily prednisone use of 10 mg or greater over 24 weeks [8]. The results revealed that over 60% of patients in the tocilizumab group achieved CRP levels less than 10 mg/dL compared to 30% in the placebo group at 24 weeks [8]. They also demonstrated that almost 50% of the patients in the tocilizumab group were able to wean off of prednisone therapy compared to less than 20% of placebo patients [8].

COVID-19 in relation to PMR and GCA

There have been various case reports in the literature regarding immune-mediated flare-ups of inflammatory disease or new-onset inflammatory disease following COVID-19 infection or administration of SARS-COV-2 vaccines. For example, one case report discussed a patient that presented with bilateral shoulder and hip stiffness, vision loss, and jaw claudication about a month following COVID-19 infection [9]. This patient had a temporal artery biopsy done which showed recanalization after inflammation. However, other imaging, such as computed tomography angiography (CTA) and magnetic resonance angiography (MRA), were negative for aortitis [9]. Another case discussed a patient that tested positive for COVID-19 infection and initially presented with bilateral temporal artery tenderness and headaches along with cough and fever, which was thought to be related to COVID-19 [10]. However, he presented with persistent headaches and jaw pain a month later. His ESR, CRP, and autoimmune profiles were negative; however, an ultrasound of the temporal artery showed the halo sign, confirming the diagnosis of GCA [10]. There are also rare reports of PMR or GCA development following SARS-COV-2 vaccination. One such report described a patient that developed PMR symptoms one day following the first dose of a Pfizer SARS-COV-2 vaccine called Tozinameran (BNT162b2), which is a nucleoside-modified messenger RNA (mRNA) vaccine [11]. This patient did not have GCA symptoms, with normal findings on Doppler ultrasound of temporal arteries and rapid improvement of symptoms with steroids. She did receive a second vaccine dose and continued treatment with low-dose prednisone a few months thereafter [11]. Another case report described a patient that developed GCA symptoms two days after receiving a SARS-COV-2 mRNA vaccine, and the diagnosis was proven through a temporal artery biopsy [3]. The mechanism of these types of associations with COVID-19 is still not well-understood. However, there seems to be an interplay between the innate and adaptive immune systems in the amplification of inflammatory pathways. Whether with infection or immunization, it is clear that some immune dysregulation occurs that allows autoimmune syndromes to form or even accelerate subclinical pre-existing autoimmune conditions [12]. One study particularly looked at the incidence of new-onset systemic rheumatic disease following either COVID-19 symptoms or a positive polymerase chain reaction (PCR) test, and they found a ratio of about 5-6 cases out of 15,000 [12]. Some of these cases included new-onset GCA, PMR, polymyositis, inflammatory arthritis, antiphospholipid syndrome (APS), and primary Sjogren’s syndrome. GCA, in particular, seems to be a common manifestation following COVID-19 infections, which may be due to the affinity of the virus for vasculopathy and hypercoagulable states [12].

## Conclusions

PMR and GCA are frequently seen rheumatologic conditions that are well-known to be associated. Ongoing research about their pathophysiology has inspired trials for effective targeted therapies. Several biologic medications continue to be studied with the increasing trend to move away from long-term glucocorticoid therapy. The association between COVID-19 infections triggering new-onset autoimmune disease is not well-understood, although a few cases of rheumatic disease have been reported following both COVID-19 infections and vaccines. This raises concern for closer monitoring of such patients at the time of their diagnosis with COVID-19 or after vaccine administration for the possibility of developing a new autoimmune disease. 
